# Spontaneous Endometrioma Rupture: A Retrospective Pilot Study and Literature Review of a Rare and Challenging Condition

**DOI:** 10.3390/jcm14103387

**Published:** 2025-05-13

**Authors:** Georgios Kolovos, Ioannis Dedes, Saranda Dragusha, Cloé Vaineau, Michael Mueller

**Affiliations:** 1 Department of Obstetrics and Gynecology, Bern University Hospital, 3010 Bern, Switzerland; d_jannis@hotmail.com (I.D.); cloe.vaineau@insel.ch (C.V.); michel.mueller@insel.ch (M.M.); 2 Faculty of Medicine, University of Bern, 3010 Bern, Switzerland; s.dragusha@spitalmaennedorf.ch

**Keywords:** ruptured endometrioma, ovarian endometriosis, acute abdominal pain, emergency laparoscopy

## Abstract

**Background/Objectives**: Endometriosis can present as ovarian endometriosis in 15–25% of the cases. While chronic pelvic pain and dysmenorrhea dominate its clinical presentation, acute complications, such as spontaneous OMA rupture, are rare (<3%), often mimicking acute abdominal pain and necessitating emergency surgery. Diagnostic delays persist due to the condition’s rarity and overlapping symptoms with ovarian torsion or appendicitis. This study investigates the clinical features of ruptured OMAs to enhance preoperative suspicion and optimize management. **Methods**: From February 2011 to August 2023, 14 patients with spontaneous rupture of histologically confirmed endometriomas underwent emergency laparoscopy for acute abdominal pain in the University Hospital of Bern, Switzerland. The clinical data of these patients were analyzed to find common patterns of spontaneous endometrioma ruptures. We also conducted a literature search in PubMed, Scopus, ScienceDirect, Cochrane, and Embase databases from inception to December 2023 in order to identify other possible confounding factors. The search was based on the keywords “ruptured endometrioma”. All English full-text prospective and retrospective observational and interventional studies with at least five patients that described the clinical features and findings of women diagnosed with ruptured endometrioma and treated surgically were included. **Results**: The median age at operation was 37.4 (23–49) years old, and all cases presented with acute abdominal pain, with/without peritonitis. Only 3/14 patients presented with fever, while the most common laboratory finding was an elevated CRP level of 45.6 mg/L (3–100 mg/L), while leukocytosis was less pronounced, with a median of 12.2 G/L (6.04–21.4 G/L). Notably, 64.3% (9 out of 14) of the patients reported experiencing dysmenorrhea, while for the remaining 5 individuals, the presence or absence of dysmenorrhea could not be obtained. Interestingly, only one patient had undergone hormonal treatment, with a combined oral contraceptive (COC) of Ethinylestradiol (0.02 mg) and Desogestrel (0.15 mg), while the other patients either lacked awareness of their endometriosis or expressed reluctance towards hormonal downregulation therapy. The median endometrioma size was 7 cm (3.5–18 cm), and 78.57% of the cases (11 out of 14 patients) had only ovarian endometriosis, while only 3 patients had involvement of compartment A, B, or C according to the # ENZIAN classification. **Conclusions**: Though rare, spontaneous OMA rupture should be considered in acute abdomen cases, especially with cysts > 5 cm. Hormonal therapy may reduce rupture risk, but more research is needed to confirm this and refine diagnostic strategies.

## 1. Introduction

Endometriosis is a chronic gynecological disorder characterized by the growth of endometrial-like tissue outside of the uterine cavity, primarily affecting the pelvic peritoneum, ovaries, uterosacral ligaments, and rectovaginal septum [1,2,3]. Clinically categorized into three subtypes—superficial peritoneal endometriosis, deep infiltrating endometriosis, and ovarian endometriomas (OMAs)—the disease impacts 10–15% of reproductive-aged women, with prevalence rising to 14–77% among those with unexplained infertility [1,4]. Common symptoms include dysmenorrhea (painful menstruation), chronic pelvic pain, dyspareunia (pain during intercourse), and infertility, alongside less frequent manifestations, such as urinary or bowel dysfunction, shoulder pain (associated with diaphragmatic involvement), and systemic fatigue [5,6]. These symptoms profoundly impair quality of life, necessitating individualized treatment strategies ranging from hormonal therapies (e.g., oral contraceptives, progestins) to surgical intervention for severe or refractory cases. Despite advances in care, recurrence rates remain high, with 40–50% of patients experiencing disease resurgence within five years of surgery [7,8].

Acute abdominal pain, defined as non-traumatic pain lasting ≤7 days, accounts for 7% of emergency department visits and poses a diagnostic challenge in women of reproductive age [9,10]. The differential diagnosis spans self-limiting conditions to life-threatening emergencies. The most common causes of acute abdominal pain in women include ovarian torsion, ectopic pregnancy, pelvic inflammatory disease, and ovarian masses, with less frequent causes, such as uterine fibroids and endometriosis, also contributing to such a clinical presentation [11]. Rupture of an ovarian cyst is a common cause of abdominal pain in women of reproductive age presenting to the emergency department. While often a self-limiting physiological process, complications, such as hemorrhage or torsion, may necessitate surgical intervention. This spectrum of cyst rupture involves functional cysts (e.g., follicular and corpus luteum) and, less commonly, non-functional cysts (e.g., teratomas, endometriomas, and cystadenomas) [12,13].

Endometriomas or ovarian endometriosis cysts are thought to develop through different mechanisms, including endometriotic invasion or metaplasia of functional cysts, or from ovarian surface endometriosis that bleeds into the ovarian cortex. These varied pathways of formation may help explain the phenotypic diversity and clinical characteristics observed among endometriomas [14]. Torsion of an endometrioma is relatively uncommon due to the presence of pelvic adhesions, which limit ovarian mobility. Similarly, rupture is rare, affecting less than 3% of diagnosed endometriomas [15].

In this study, we investigate the clinical features of spontaneous OMA rupture through a cohort of patients presenting with acute abdomen pain. By synthesizing preoperative findings, surgical outcomes, and literature insights, we aim to enhance diagnostic accuracy and optimize management strategies for this underrecognized complication of endometriosis.

## 2. Materials and Methods

In this retrospective study, we reviewed histopathological records of patients diagnosed with ovarian endometriosis (OMAs) at the SEF (Stiftung Endometriose Forschung)-certified Endometriosis Center of Bern University Hospital. We then analyzed surgical reports to identify cases that underwent emergency laparoscopy, excluding elective procedures. Between February 2011 and August 2023, 14 such patients were identified. All patients presented with sudden onset of abdominal pain and underwent emergency laparoscopy. Surgical pathology confirmed the diagnosis of endometriomas in each case. This study was approved by the institutional review board of the University Hospital of Bern. The inclusion criteria for this study were premenopausal patients aged 15 years and older. Patients were excluded if they were pregnant, breastfeeding, or had a diagnosis of malignant disease. 

**Figure 1:**
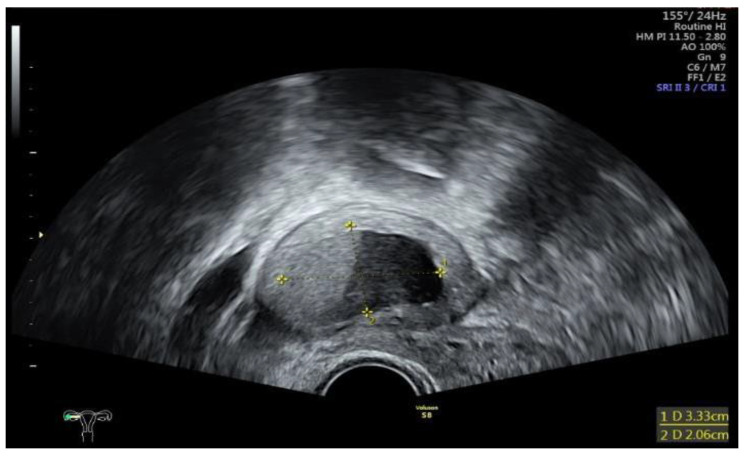
Sonographic display of a ruptured endometrioma of the right ovary in a 28-year-old woman with sudden onset of abdominal pain.

**Figure 2:**
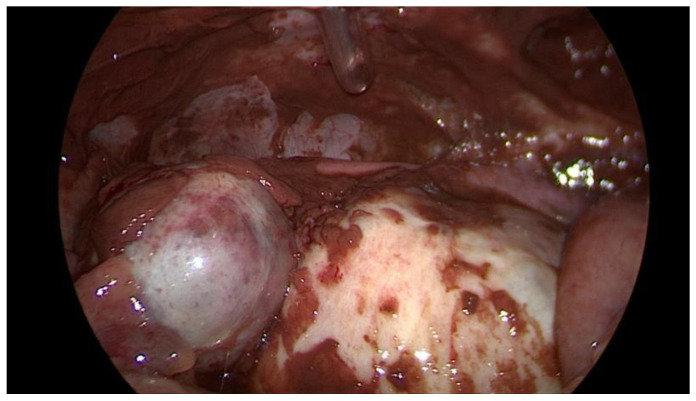
Intraoperative laparoscopic display of a ruptured endometrioma of the left ovary in a 43-year-old woman.

**Figure 3:**
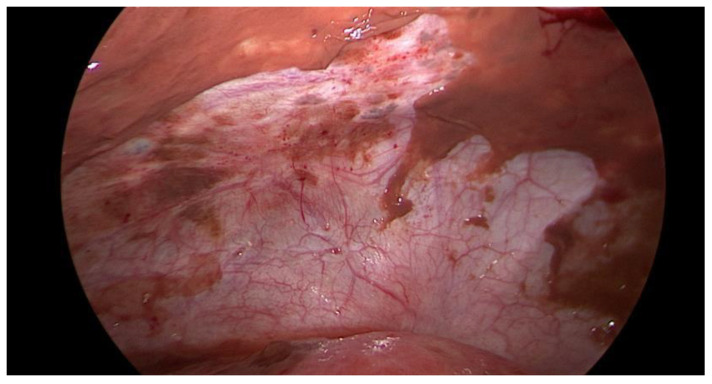
Intraoperative laparoscopic display of anterior pelvic spillage in a patient with concomitant peritoneal endometriosis.

Data collection: The patients’ backgrounds, clinical symptoms at admission, sonographic and laboratory assessment, as well as intraoperative findings were recorded. Patient demographic data included age, menopausal status, presence of dysmenorrhea, history of endometriosis surgery, and use or non-use of hormonal downregulation therapy for endometriosis. Pre-operative laboratory tests included white blood cell (WBC) count, serum C-reactive protein (CRP) level, and serum CA125 level, and all patients underwent transvaginal sonography (Figure 1) on admission as part of the emergency diagnostic assessment. The maximal diameter and the location of the endometriomas were noted. The intraoperative findings included the location of the ruptured endometriomas (Figure 2 and Figure 3), the presence and classification of endometriosis according to the rASRM [16] and #ENZIAN [17] systems, and, lastly, total blood loss.

**Figure 4:**
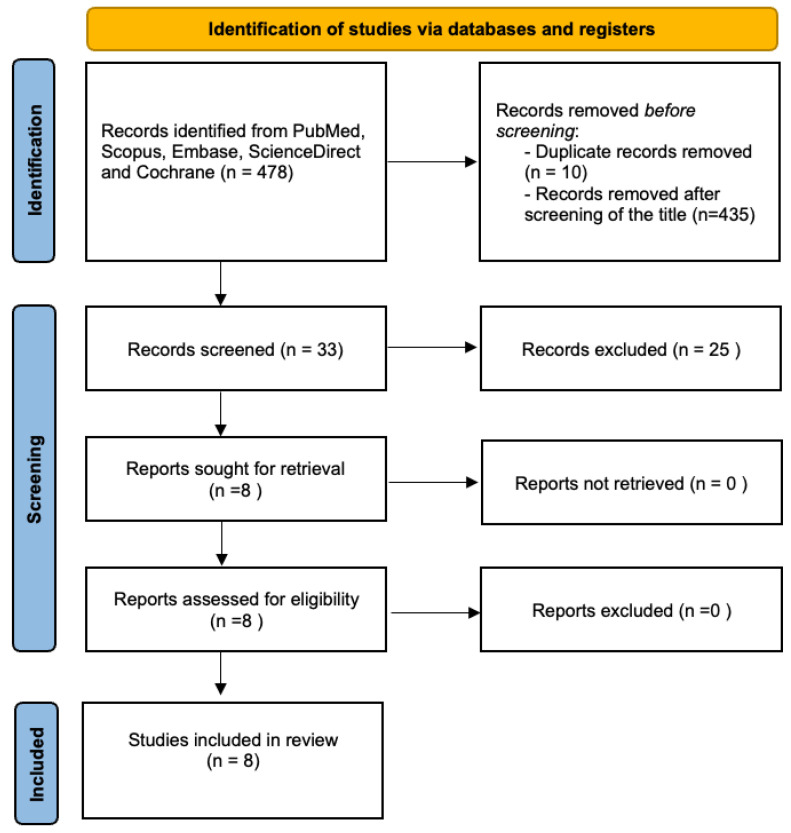
Flowchart for study selection according to the PRISMA guidelines.

We then conducted a literature review using the PubMed, Scopus, ScienceDirect, Cochrane, and Embase databases from inception to December 2023 with the keyword “ruptured endometrioma”, as illustrated in the flowchart (Figure 4). The review was performed in accordance with the guidelines of the Preferred Reporting Items for Systematic Reviews and Meta-Analysis (PRISMA). The initial search identified 478 articles. After title screening and removing duplicates, eight records were included in the final analysis. The inclusion criteria for study selection encompassed cohort studies, case-control studies, and randomized controlled trials (RCTs) published within the last 30 years. Only articles published in English were considered. The population of interest included premenopausal and postmenopausal women who underwent laparoscopy, either elective or emergency, with a confirmed diagnosis of a ruptured endometrioma.

## 3. Results

The demographic data and preoperative and intraoperative characteristics of the 14 patients are presented in Table 1, while information on endometriosis-related symptoms, previous endometriosis surgeries, and intraoperative endometriosis classification is detailed in Table 2.

The median age at operation was 37.8 (±7.2) years old, and all patients were premenopausal. All cases presented to the hospital with acute persisting abdominal pain with/without peritonitis, and only 3/14 (21.4%) patients presented with fever. The most common laboratory finding was a high CRP level of mean 45.6 mg/L (<3–100 mg/L), while the leucocytes were not significantly elevated, with a mean level of 12.2 G/L (6.04–21.4 G/L). Serum levels of the tumor marker CA-125 were measured in only two patients. Therefore, no conclusions can be drawn.

Nine out of fourteen (64.3%) of the patients reported experiencing dysmenorrhea, while for the remaining five individuals, the presence or absence of dysmenorrhea could not be obtained. Interestingly, only one patient had undergone hormonal treatment before surgery, involving continuous uptake of Ethinylestradiol (0.02 mg) and Desogestrel (0.15 mg), while the rest of the patients either lacked awareness of their endometriosis or expressed reluctance towards hormonal downregulation therapy. The median endometrioma size was 7.8 cm (±4 cm, 3.5–18 cm). Five out of fourteen patients (35.7%) had endometrioma on the right ovary, six out of fourteen (42.9%) had endometrioma on the left, and three out of fourteen patients (21.4%) had bilateral endometriomas. The vast majority of the patients had only ovarian endometriosis, with a prevalence of 78.57% (11 out of 14 patients), while only 3 patients had involvement of compartment A, B, or C according to the # ENZIAN classification. One patient had deep infiltrating endometriosis of the bladder (FB according to the #ENZIAN), and another one of the intestine (Sigmoid colon, FI according to the #ENZIAN). Six out of fourteen patients (42.9%) had endometriosis grade I according to the revised American Society for Reproductive Medicine (rASRM) score, five out of fourteen (35.7%) had grade II, one out of fourteen (7.1%) had grade III, and two patients (14.3%) had grade IV.

As far as reviewing the literature is concerned, eight studies were included in the final analysis. Tanaka et al. [18] from Korea conducted a retrospective study comparing six cases of ruptured endometriomas requiring emergency surgical treatment with 16 patients in a control group who underwent elective surgical removal of their endometriomas. The study found that plasma D-dimer levels were significantly higher in cases of ruptured endometriomas compared to unruptured cases. All patients with ruptured endometriomas had preoperative plasma D-dimer levels exceeding 1.5 mg/mL, whereas none of the unruptured cases had levels above 1.0 mg/mL. Statistically significant differences were noted in the white blood cell (WBC) count, with normal values in the control group compared to 10.8 ± 3.4 (× 1000/mL) in the study group. Similarly, elevated C-reactive protein (CRP) levels were observed in the study group, with a mean value of 9.4 ± 5.4 mg/dL, indicating a heightened inflammatory response associated with endometrioma rupture. The tumor marker CA125 level was not significantly different between the two groups.

The results of this study do not, however, align with the findings of the study by Dai et al. [19]. In this retrospective study from China, 43 patients with spontaneous endometrioma rupture were compared with 70 women with unruptured ovarian endometriomas, who were selected as a control group for comparative analysis. Tumor marker levels, as well as the WBC and CRP levels and the size of the endometrioma, were evaluated. CEA (Carcinoembryonic antigen) and AFP (alpha-fetoprotein) levels showed no significant differences between the two groups. However, the mean values of CA-125 (cancer antigen) and CA19-9 (Carbohydrate antigen) levels were significantly higher in the study group. CA-125 levels had a mean value of 797.89 ± 1106.52 (U/mL) in the study group versus 46.43 ± 39.61 (U/mL) in the control group. Similarly, CA 19-9 was 1058.07 ± 2262.49 (U/mL) in the study group versus 31.91 ± 48.18 (0.8–306.0) (U/mL) in the control group. The white blood cell (WBC) counts were also significantly higher in the ruptured group, with values of 8.85 ± 4.57 (×1000/mL). The median CRP level was notably elevated in the study group at 72.5 mg/dL. In contrast, CRP levels were not routinely measured in patients undergoing elective surgery for unruptured endometriomas. According to Huang et al. [20], a ruptured ovarian endometrioma should be highly suspected in the following cases: the presence of free fluid in the pouch of Douglas with or without an ovarian mass, sudden onset of abdominal pain, a surgical history of endometriomas, or clinical suspicion of endometriosis. In another retrospective study by the same research group [21], which examined 43 patients with ruptured endometriomas who underwent emergency surgical intervention, it was found that 81.6% of the patients with cyst rupture had a known ovarian endometrioma, with an average size greater than 6 cm prior to rupture [21]. Similarly, Gu et al. [22], in a larger, retrospective study involving 38 patients with ruptured endometriomas and 76 patients in the control group with unruptured endometriomas, found that the maximum diameter of the OMA in the study group was significantly larger than in the unruptured group (7.3 ± 1.7 vs. 6.6 ± 1.9). Patients with ruptured endometriomas also had a significantly lower BMI (body mass index) and a higher proportion of cul-de-sac partial obliteration, as opposed to complete obliteration (*p* = 0.003). The r-ASRM score in the unruptured group was higher than in the ruptured group, although the difference was not statistically significant (52.6 ± 25.8 vs. 44.6 ± 22.5, *p* = 0.089). In our study, we also found that the vast majority of patients had r-ASRM scores of I–II, rather than III or IV.

A Corean retrospective study [23] involving 14 patients with ruptured endometriomas and 60 patients with unruptured endometriomas investigated preoperative diagnostic indicators that can help in diagnosing ruptured endometriomas. There were no significant differences in the background demographic characteristics between the two groups in terms of age, parity, last menstrual cycle days, or the median size of the endometriomas. However, a notable difference in serum markers was observed. The median CA 125 level was significantly higher in the study group, with median levels of 345.1 U/mL compared to 49.8 U/mL in the control group. Similarly, the median CA 19-9 level was 46.0 U/mL in the ruptured endometrioma group versus 19.1 U/mL in the non-ruptured group. CRP levels were also markedly elevated, with a median of 12 mg/L versus 3 mg/L in Group B (*p* = 0.000). Receiver operating characteristic (ROC) analysis identified optimal cutoff values for predicting rupture: 100.9 U/mL for CA 125, 27.7 U/mL for CA 19-9, and 10 mg/L for CRP. In a similar methodological study by Turkey [24], 35 patients undergoing surgery for ruptured endometriomas were compared to 146 patients undergoing elective surgery for non-ruptured endometriomas. Preoperative levels of CRP, mean platelet volume (MPV), and tumor markers CA 125, CA 19-9, CA 15-3, and CEA, as well as postoperative MPV and neutrophil-to-lymphocyte ratio (NLR), were compared. It was found that all of these parameters were significantly higher in the ruptured endometriomas group compared to the control group. In contrast, postoperative lymphocyte and eosinophil levels were significantly lower in the study group. When evaluating preoperative biomarkers for predicting rupture, MPV (mean platelet volume), CA 19-9, and CA 15-3 demonstrated high specificity (>75%) but low sensitivity (<60%), while CRP, CA 125, and CEA showed high sensitivity but low specificity. The final study included in our literature review focused on comparing CT findings of ruptured endometriomas with those of ruptured ovarian functional cysts [25]. The primary objective was to enhance and optimize the differential diagnosis in cases where cyst rupture is clinically suspected. This study retrospectively analyzed 13 patients with surgically confirmed ruptured endometriomas and 25 cases of surgically confirmed ruptured ovarian functional cysts who underwent preoperative CT scanning. The mean maximum diameter of endometriotic cysts (70.1 mm) was significantly larger than that of functional cysts (36.4 mm). Preoperative CT scanning demonstrated significantly higher percentages of peritoneal stranding and infiltration in cases of ruptured endometriotic cysts rather than ruptured follicular cysts. Furthermore, endometriomas more frequently exhibited a multilocular structure and thicker cyst walls compared to functional cysts, but, after contrast agent application, extravasation at the bleeding site was observed in 11 out of 25 functional cyst cases but only 1 out of 13 endometriotic cyst cases. Ascites, when present, is also more commonly located to the pelvic cavity in cases of ruptured endometriomas.

## 4. Discussion

Spontaneous rupture of ovarian endometriomas (OMAs) presents a clinical challenge due to its nonspecific presentation, often mimicking other causes of acute abdomen pain, such as ovarian torsion, appendicitis, or ectopic pregnancy. In our cohort, all patients underwent emergency laparoscopy, reflecting the universal need for prompt surgical intervention regardless of the underlying etiology. This aligns with the existing literature, where laparoscopy serves as the cornerstone for both diagnosis and management of acute abdominal pain in reproductive-aged women. While the immediate surgical approach—cystectomy, adhesiolysis, and peritoneal lavage—remains consistent across conditions like torsion and rupture, long-term management diverges significantly. Unlike ovarian torsion, which focuses on preserving ovarian function and addressing anatomical predispositions, OMA rupture necessitates comprehensive endometriosis staging (e.g., rASRM/#ENZIAN classification) and postoperative hormonal therapy to mitigate recurrence. This distinction underscores the importance of intraoperative documentation of endometriosis severity to guide tailored postoperative strategies, such as hormonal suppression with progestins or combined oral contraceptives (COCs).

**Figure 5:**
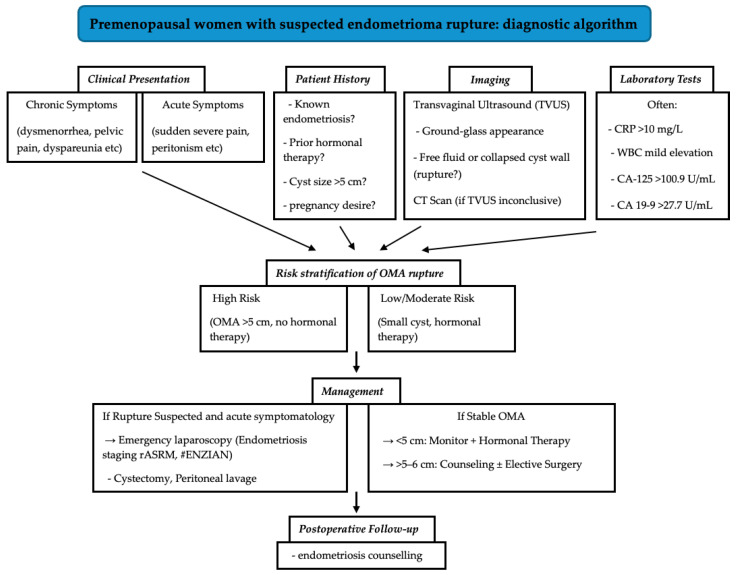
Diagnostic algorithm for premenopausal patients with suspected ovarian endometrioma rupture.

A critical insight from our study is the striking prevalence of untreated endometriosis among patients with ruptured OMAs, as 92.9% had not received hormonal therapy prior to rupture, primarily due to lack of disease awareness or reluctance towards treatment due to side effects of the hormonal treatment. This suggests a potential association between untreated endometriosis and rupture risk, particularly in larger cysts (>5 cm). These findings necessitate a paradigm shift in patient counseling. Individuals with endometriomas, especially those with cysts exceeding 5 cm, should be informed that rupture can occur even in the absence of prior symptoms or endocrine treatment. Counseling must emphasize the importance of regular monitoring for asymptomatic endometriosis and the role of hormonal agents in reducing—but not abolishing—risk. For patients with endometriosis who are unable or unwilling to use hormonal downregulation treatments due to adverse effects, it is essential to explore alternative management strategies. Given the wide variety of hormonal treatment options available—with differing mechanisms, dosages, and side effect profiles—it is reasonable as a first step to explore alternative hormonal therapies that may be better tolerated before abandoning this approach entirely. If this is not possible, non-hormonal options, such as non-steroidal anti-inflammatory drugs (NSAIDs), can be effective in managing the painful symptoms associated with endometriosis, while complementary and alternative therapies, including acupuncture, dietary changes, and physiotherapy, should also be offered. To translate our findings into clinical practice, we propose a diagnostic algorithm that integrates patient history, symptomatology, imaging, and laboratory markers to guide the evaluation and management of suspected ovarian endometrioma (Figure 5). 

Our study is limited by its retrospective design and small sample size. This limits our ability to draw causal conclusions due to the lack of statistical power of our analysis. Larger prospective studies are needed to validate the prophylactic role of hormonal therapy, standardize diagnostic algorithms integrating clinical and imaging data, and explore patient adherence to postoperative regimens. Nevertheless, the clinical implications are clear. While the acute management of OMA rupture mirrors that of ovarian torsion, the underlying etiology demands distinct long-term strategies.

The literature review reveals conflicting evidence on diagnostic biomarkers. While some studies report elevated D-dimer and CRP levels in ruptured cases, others highlight significantly higher CA-125 and CA19-9 levels. These discrepancies underscore the limitations of relying solely on biomarkers for diagnosis. Instead, imaging modalities, such as CT scans, offer valuable adjunctive insights. For instance, ruptured endometriomas are often larger (>6 cm) and exhibit features like peritoneal stranding or multilocular structures, distinguishing them from functional cysts. Such findings, combined with acute clinical symptoms, should heighten suspicion for rupture and prompt urgent surgical evaluation. In conclusion, enhanced clinician awareness, proactive patient education, and individualized follow-up are essential to reducing the morbidity associated with this rare but serious complication.

## 5. Conclusions

This study underscores that spontaneous rupture of ovarian endometriomas (OMAs), though rare, poses a significant diagnostic challenge due to its mimicry of acute abdomen conditions, such as ovarian torsion. A striking 92.9% of ruptured OMA cases occurred in patients untreated with hormonal therapy, highlighting a potential association between untreated endometriosis and rupture risk, particularly in larger cysts (>5 cm). These findings underscore the importance of routine monitoring for cysts exceeding 5 cm and timely hormonal therapy counseling to mitigate rupture risk. Despite limitations from the small sample size and the retrospective design, these findings advocate for heightened clinical vigilance, individualized postoperative strategies, and larger prospective studies to validate hormonal prophylaxis and refine diagnostic algorithms. Enhanced awareness and proactive patient education are critical to reducing morbidity in this underrecognized complication of endometriosis.

## Figures and Tables

**Table 1:** Preoperative and intraoperative characteristics.

Patient	Age at Surgery	Menopause 0: No, 1: Yes	CA 125 (U/mL)	CRP (mg/L) at Surgery	Lc (G/L) at Surgery	Fever at Admission	Bilateral	Left	Right	Size in cm (Diameter) of the Largest Lesion	Blood Loss
1	28	0	n/a	<3	13.3	0	0	0	1	4.8	minimal
2	39	0	n/a	n/a	6.04	0	0	1	0	n/a	200 mL
3	43	0	n/a	100	n/a	0	0	0	1	7.2	minimal
4	43	0	n/a	86	21.4	0	0	1	0	18	500 mL
5	36	0	n/a	n/a	9.1	0	0	1	0	n/a	2.500 mL
6	36	0	1125	22	n/a	0	0	1	0	7	300 mL
7	49	0	n/a	96.8	13.7	0	0	0	1	n/a	300 mL
8	34	0	n/a	<3	7.61	0	1	1	1	7.5	minimal
9	40	0	n/a	28	14	0	0	0	1	8	minimal
10	49	0	n/a	52	12.5	1	1	1	1	4.8	200 mL
11	39	0	41.6	n/a	n/a	0	0	1	0	5	n/a
12	32	0	n/a	27	n/a	1	0	0	1	n/a	n/a
13	23	0	n/a	n/a	n/a	1	1	1	1	4	minimal
14	33	0	n/a	n/a	n/a	0	0	1	0	3.5	200 mL

n/a: not available.

**Table 2:** Endometriosis characteristics.

Patient	Age at Surgery	Dysmenorrhea Yes = 1	Prior Endometriosis Surgery (Yes = 1, No = 0)	Number of Prior Endometriosis Surgeries	Hormonal Therapy Before Surgery: (Yes = 1, No = 0)	rASRM I	rASRM II	rASRM III	rASRM IV	#ENZIAN Score
1	28	1	0	0	0	0	1	0	0	O0/2 B1 FB
2	39	n/a	1	1	0	0	1	0	0	O1/0 A2 FA
3	43	n/a	1	2	0	1	0	0	0	O0/3
4	43	n/a	0	0	0	1	0	0	0	O3/0
5	36	n/a	0	0	0	1	0	0	0	P1 O1/0
6	36	1	1	0	0	1	0	0	0	O2/0
7	49	1	1	1	0	1	0	0	0	O0/n/a
8	34	1	0	0	0	0	1	0	0	O1-2/3
9	40	1	1	2	0	0	0	0	1	P3 O3/0 C1
10	49	n/a	0	0	0	0	0	0	1	O2/2 FI
11	39	1	0	0	0	0	1	0	0	O2/0
12	32	1	1	1	1	1	0	0	0	O0/n/a
13	23	1	0	0	0	0	1	0	0	O2/2
14	33	1	0	0	0	0	0	1	0	O2/0

n/a: not available.

## Data Availability

Data sharing is not applicable to this study because of privacy or ethical restrictions.
